# Gut microbiota dysbiosis impairs TGF-β/Smad4 signaling to drive postoperative metastasis in colorectal cancer

**DOI:** 10.3389/fmicb.2025.1654227

**Published:** 2025-12-15

**Authors:** Tongtong Lan, Qilong Hou, Huichao Zhao, Yiman Luan, Yuying Shi, Shengrui Hu, Ningrui Wang, Shiyu Yan, Xinyi Gong, Yang Song

**Affiliations:** 1 College of Medicine, Qingdao University, Qingdao, China; 2 School of Public Health, College of Medicine, Qingdao University, Qingdao, China; 3 Qingdao Municipal Hospital, University of Health and Rehabilitation Sciences, Qingdao, China; 4 School of Pharmacy, College of Medicine, Qingdao University, Qingdao, China; 5 Qingdao Cancer Institute, The Affiliated Hospital of Qingdao University, Qingdao University, Qingdao, China

**Keywords:** colorectal cancer, gut microbiota, metastasis, postoperative patients, SCFAs

## Abstract

**Background:**

After surgical intervention, metastasis remains the primary contributor of mortality rates in colorectal cancer (CRC). While compelling evidence implicates gut microbiota dysbiosis as a key driver of CRC progression, its role in postoperative metastasis remains unclear.

**Methods:**

A total of 97 participants were recruited, comprising 21 postoperative CRC patients with metastasis (M group), 37 postoperative CRC patients without metastasis (C group), and 39 healthy individuals (H group). Fecal microbiota composition, short-chain fatty acid (SCFA) concentrations, and serum cytokines were quantified. Spearman correlation was used to assess the relationship between microbiota and SCFAs. Fecal microbiota transplantation (FMT) was performed by transferring patient specimens into antibiotic-pretreated orthotopic CRC mice models. Epithelial-mesenchymal transition (EMT) markers and TGF-β/Smad4 signaling were subsequently analyzed.

**Results:**

In humans, M group patients exhibited significant gut dysbiosis, characterized by enriched *Fusobacterium* and depleted *Gemmiger*, concomitant with markedly diminished fecal butyrate, propionate, and acetate (vs. H group, *p* < 0.05). In these patients, this dysbiosis was directly correlated with SCFA depletion (*P_adj_* < 0.05). Serologically, patients in the M group exhibited elevated TGF-β while suppressed IL-10 compared to C group (*p* < 0.05). In the mouse model, the FMT from M group significantly increased *Fusobacterium* abundance and reduced fecal acetate/butyrate, concomitantly accelerating tumor progression with elevated hepatic and cecal tumor weights and upregulated EMT markers (N-cadherin and MMP9). This pro-metastatic phenotype was associated with downregulated hepatic mRNA expression of the key SCFA receptors, FFAR2 and FFAR3. Notably, in the animal model, the FMT-M group exhibited elevated hepatic TGF-β and a trend toward reduced Smad4 expression, suggesting a potential dysregulation of the TGF-β/Smad4 signaling pathway. Conversely, the fecal microbiota from C group markedly suppressed *Fusobacterium* colonization and restored acetic acid, butyric acid levels which ameliorates pathological changes by attenuating N-cadherin expression and normalizing TGF-β/Smad4 signaling.

**Conclusion:**

Gut microbiota dysbiosis and SCFA depletion exert profound regulatory effects on postoperative CRC metastasis, potentially by promoting EMT through mechanisms involving the TGF-β/Smad4 signaling axis. Thus, targeting gut microbiota may offer promising therapeutic strategies to mitigate CRC metastasis.

## Graphical abstract

**Figure:**
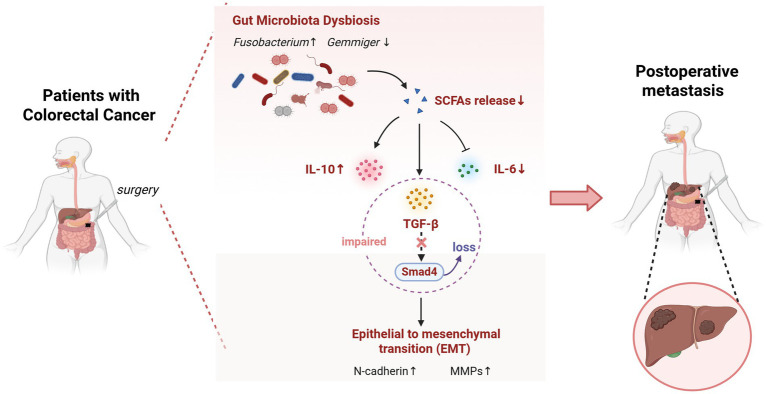

## 1 Introduction

Colorectal cancer (CRC), a prevalent malignancy within the gastrointestinal tract, is globally the third leading cause of cancer incidence and the second contributor to cancer-related death (Bray et al., 2024). Despite curative resection followed by adjuvant radiotherapy and/or chemotherapy, approximately 50–60% of Stage II/III CRC patients still experience local recurrence or distant metastases. Consequently, the 5-year survival rate for these patients plummets to just 14% (Biller and Schrag, 2021; Neumann et al., 2010; Lambdin et al., 2023). Hence, deploying efficacious strategies to curb metastatic progression following surgery is critical for patient survival and quality of life.

Mounting evidence implicates gut microbial dysbiosis as a pivotal factor in the pathogenesis and progression of CRC, which is characterized by diminished microbial diversity, restructuring of microbial community composition, and concomitant perturbations in metabolic profiles (Flemer et al., 2017; Wong and Yu, 2019; Shah et al., 2018; Nakatsu et al., 2015; Chen et al., 2022; Bullman et al., 2017). Distinct microbiome shifts have been revealed across CRC stages—from multiple polypoid adenomas (MP) and intramucosal carcinomas (Stage 0) to Stage I/II and Stage III/IV—relative to healthy controls, demonstrating marked stage-specific variations (Feng et al., 2015; Yachida et al., 2019). Specifically, the relative abundance of *Fusobacterium nucleatum* spp. exhibits a progressive elevation from S0, SI/II and SIII/IV, increasing with the degree of malignancy. In addition, patients who have undergone curative resection of adenomas exhibit distinct gut microbiota alterations versus healthy controls, characterized by reduced *Bifidobacterium* and butyrate-producing bacteria (Yachida et al., 2019). Collectively, these findings indicate that the gut microbiota not only undergoes dynamic alterations across CRC progression stages, but also maintains significant distinct deviations in post-surgical patients compared to healthy individuals.

Alterations in the structure of the gut microbiome, especially the enrichment of pathogenic bacteria and depletion of beneficial bacteria, can lead to changes in the tumor microenvironment, in which the change of EMT related markers are most commonly reported (Fearon and Vogelstein, 1990). EMT cascade is a critical step in tumor metastasis which frequently accompanied with loss of E-cadherin expression and increased N-cadherin, VEGF, and MMP-9 expressions which prompt carcinoma metastasis and colonization (Thiery, 2002). Research indicates that *F. nucleatum* can invade CRC cells and release the FadA adhesion, which binds to E-cadherin, thereby promoting the EMT process (Rubinstein et al., 2013). Probiotics such as *Bifidobacterium* can mediate the EMT process through IL-6, thereby inhibiting the invasion and metastasis of HCT116 cells (Du et al., 2022).

The progression of CRC is shaped by a complex interplay among the gut microbiota, the intestinal barrier, the immune system, and microbial metabolites like short-chain fatty acids (SCFAs) (Karam et al., 2025). Within this axis, inflammatory cytokines emerge as critical mediators orchestrating the EMT process, and their production is intricately regulated by the gut microbiota. For instance, specific bacteria such as *F. nucleatum*can directly stimulate tumor and immune cells to produce pro-inflammatory cytokines like IL-6 and TGF-β, which are known drivers of EMT and metastasis (Rubinstein et al., 2013; West et al., 2015). A large-scale integrative analysis in colorectal adenoma revealed that *F. nucleatum* enrichment is strongly correlated with markers of inflammatory signaling and stem cell proliferation (Ma et al., 2025). Conversely, beneficial bacteria and their metabolites, particularly short-chain fatty acids (SCFAs), can modulate the immune environment by promoting the production of anti-inflammatory cytokines like IL-10 or by directly influencing TGF-β signaling (Shouval et al., 2014; Bauché and Marie, 2017). TGF-β itself plays a dual role; it acts as a tumor suppressor in early-stage cancer but promotes invasion and metastasis in advanced stages (Calon et al., 2012). Despite these connections, the specific cytokine profiles in postoperative CRC patients and how they are shaped by gut microbiota dysbiosis to influence metastatic outcomes remain poorly understood.

In this study, we hypothesized that the gut microbiota of postoperative patients with Stage II/III CRC also has its specificity. Targeting the gut microbiota represents a promising strategy to prevent metastasis in postoperative CRC patients. Nevertheless, the potential association between postoperative CRC metastasis and gut microbiota remains unresolved. This study delineates gut microbiota signatures and SCFA profiles linked to postoperative CRC metastasis, and elucidates their regulatory effects on pathological progression, thereby providing mechanistic insights essential for developing microbiota-targeted therapies to inhibit metastasis in postoperative CRC patients.

## 2 Materials and methods

### 2.1 Participants recruiting

Individuals undergoing routine check-ups and postoperative CRC patients at the Affiliated Hospital of Qingdao University between July 2020 and December 2021 were screened based on the inclusion and exclusion criteria.

#### 2.1.1 Inclusion criteria

(1) The postoperative metastasis of CRC (M) group consists of patients with primary Stage II/III CRC who received a new diagnosis of metastatic disease within 6 months to 2 years after undergoing radical resection surgery; (2) The postoperative non-metastasis group of CRC (C group) includes patients with primary Stage II/III CRC who received curative surgery and did not develop metastasis within 2 years postoperatively; (3) The healthy controls (H group) comprise individuals without a history of gastrointestinal diseases or a family history of gastrointestinal diseases, who were undergoing routine physical check-ups.

#### 2.1.2 Exclusion criteria

(1) Individuals who are older than 75 years; (2) Individuals who developed metastasis within 6 months after curative surgery for CRC or during radio/chemotherapy; (3) Individuals with other gastrointestinal diseases, such as those with Crohn’s disease, ulcerative colitis, pigmented polyposis syndrome, or familial adenomatous polyposis; (4) Individuals having CRC in a first-degree relative; (5) Patients who have already developed distant metastasis or have severe conditions; (6) Patients with other malignant tumors or diseases affecting survival; (7) Individuals with a history of chronic diarrhea, chronic constipation, diabetes, or autoimmune diseases; (8) Individuals with lactose intolerance; (9) Individuals who have used antibiotics, radio/chemotherapy, immunotherapy, or probiotics, or other gut microbiota preparations within 1 month before sampling; (10) Individuals with a history of abdominal surgery for reasons other than CRC; (11) Pregnant women or patients with psychiatric diseases; (12) Individuals who decline to take part in the study and refuse to sign the informed consent form.

### 2.2 Samples collection from participants

Venous blood was collected before breakfast, then centrifuged at 4 °C for 10 min at 3000 rpm, and the serum was aliquoted into 1 mL microtubes. Stool samples from participants were collected and immediately frozen. All serum and stool samples were stored at −80 °C. It is important to note that sample collection from patients occurred when no invasive examinations, treatments, or interventions had been performed recently.

### 2.3 Collection of clinical data from participants

Demographic parameters, including age, BMI, gender, smoking history, and alcohol consumption, were collected via questionnaires. Pathological characteristics, comprising TNM stage (II/III) and primary tumor location, were ascertained from surgical pathology records.

### 2.4 Cell culture, lentiviral vector and infection

The mouse colon cancer cell line, CT-26, was acquired from the ATCC and cultivated in RPMI 1640 complete medium (Meilunbio, China) with 5% CO_2_ at 37 °C. The medium was supplemented with 10% (vol/vol) fetal bovine serum (FBS) (Biological Industries, Israel) and 1% penicillin/streptomycin (Meilunbio, China). A lentiviral vector carrying a dual green fluorescent protein (GFP)-luciferase (LUC) reporter (Hanbio, China) was used to infect CT-26 cells. The infection efficiency was assessed 24 h post-incubation using an inverted fluorescence microscope (related results was shown in Supplementary Figure S1). Following infection, the cells were subjected to a two-week selection with puromycin to obtain cell lines that stably express the fluorescent protein. Subsequently, cells that had traversed the Transwell chamber to the lower compartment were collected and further cultured to establish a cell line with high migratory properties. We designated this stable, highly migratory CT-26 cell line carrying a GFP/LUC vector as CT-26-H-LUC.

### 2.5 Experimental model and treatment

Male BALB/c mice weighing 20 ± 2 g at 6 weeks of age were acquired from specific-pathogen-free (SPF) (Beijing) Biotechnology Co., Ltd. (License: SCXK [Jing], 2019-0010). All mice were kept in SPF settings with a 12 h/12 h light/dark cycle at 24 ± 2 °C, and they were given free access to food and drink.

All mice were given antibiotics for 2 weeks via drinking water that contained 0.2 g/L ampicillin, neomycin, and metronidazole, as well as 0.1 g/L vancomycin. The efficacy of the antibiotic cleaning was assessed through Gram staining (related results was shown in Supplementary Figure S2). An orthotopic mouse model of CRC was established by injecting a suspension of 1 × 10^6^ CT26-H-LUC cells into the cecum’s serosal layer after the final dose of antibiotic treatment. The mice received anesthesia with 0.1% pentobarbital sodium (80 mg/kg of body weight). The quantity of injected CT26-H-LUC cells had been ascertained using the IVIS Spectrum *in vivo* imaging system (related results was shown in Supplementary Figure S3).

One week after modeling, mice were administered D-luciferin potassium (10 μL/g of body weight) (Meilunbio, China) intraperitoneally and allowed 15 min for the substance to take effect. The mice were then anesthetized with 1% pentobarbital sodium. Subsequently, IVIS Spectrum imaging system (PerkinElmer) was utilized to monitor LUC signals. The presence of a distinct fluorescent signal in the abdominal region of the mice was considered as a confirmation of successful modeling.

### 2.6 FMT experiment

We prepared the stool water for gavage in model mice using Yu et al.’s method (Wong et al., 2017). Samples were randomly chosen and mixed at equal weight from the following groups: five from postoperative metastasis patients of CRC (M group), five from postoperative non-metastasis patients of CRC (C group) and five from healthy individuals (H group). We then suspended 1 g of the mixed stool in 5 mL of normal saline. A 200 μL aliquot of the suspension was gavaged into each mouse.

**Figure 1:**
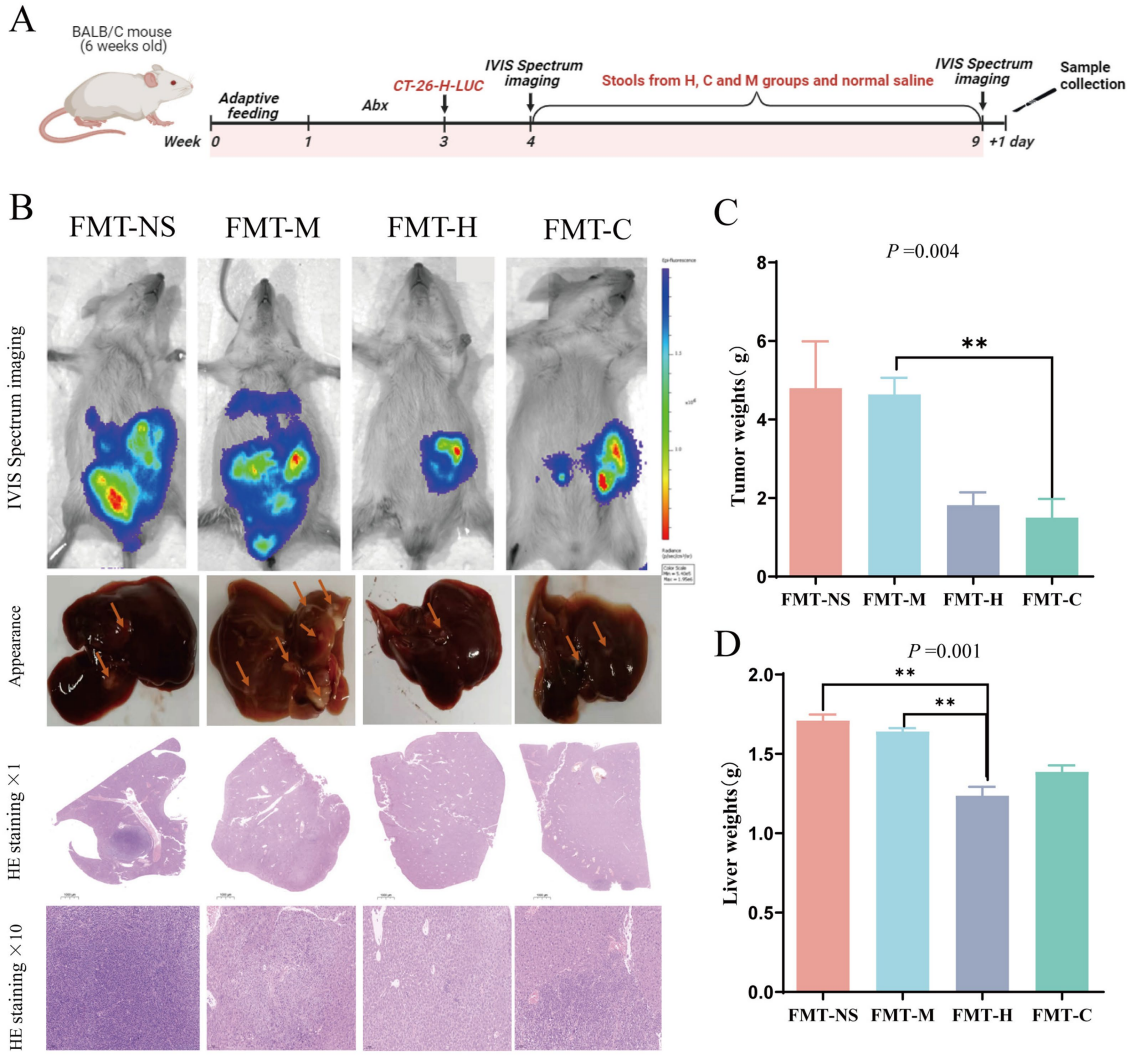
Effects of FMT on survival and tumor progression in an orthotopic CRC mouse model. **(A)** Design of stool gavage experiment to antibiotics-treated CRC orthotopic model mice. **(B)** *In vivo* imaging using the IVIS Spectrum system (prior to sacrifice), representative images of liver appearance and H&E staining sections; the slices were ×1 magnification and ×10 magnification. **(C,D)** Difference of tumor and liver weights for four groups (*n* = 3–5 per group). Values are expressed as mean ± SEM; the Kruskal–Wallis test followed by Dunn’s *post-hoc* test was used for non-parametric data. *p < 0.05, **p < 0.01, ***p < 0.001.

Based on body weight, mice that were able to detect LUC signals were split up into four groups, each consisting of 12 mice. These groups were designated as FMT-M, FMT-C, FMT-H, and FMT-NS, representing FMT from metastasis (M), non- metastasis (C), healthy (H) individuals, and a non-stool control with normal saline (NS), respectively. Each group underwent gavage with the respective stool suspension or normal saline three times per week for a duration of 5 weeks. The intervention process in animal experiments is shown in the Figure 1A.

Twelve hours following the final administration of treatment, the presence and progression of tumor metastases were assessed through *in vivo* LUC signal detection, utilizing the IVIS Spectrum imaging system. Given the technical challenges in accurately quantifying diffuse metastatic lesions via imaging or nodule counting, a composite assessment including liver weight, primary tumor weight, and mortality was employed as the most robust and unbiased measure of overall tumor burden and progression in this model. Stools of mice were collected before sacrificed, and immediately stored at −80 °C. Liver and tumor tissue *in situ* were rapidly dissected, then weighted. To be examined histopathologically, liver lobes with metastasized tumors were preserved in a 10% formaldehyde solution. The remaining tissue was promptly kept for further tests at −80 °C.

### 2.7 Enzyme-linked immunosorbent assay (ELISA)

ELISA kits (BOSTER, China) were used to measure the levels of serum TGF-β, IL-6, IL-10, and IL-17 of participants and BALB/c mice (*n* = 4), respectively, in accordance with the manufacturer’s instructions. For liver tissue analysis, a portion of the liver was homogenized in RIPA buffer and centrifuged. The resulting supernatant was collected, and its total protein concentration was determined using a BCA assay. TGF-β levels in the supernatant were then measured by ELISA in accordance with the manufacturer’s instructions.

### 2.8 DNA extraction and 16S rRNA gene sequencing and analysis

16S rRNA sequencing was performed on participant and mouse fecal samples to ascertain the composition of the gut microbiota. Total DNA extraction was performed using the CTAB method. The concentration and purity of the extracted DNA were evaluated using 1% agarose gel electrophoresis. The V3 ~ V4 region of the 16S rRNA gene was amplified via PCR with a thermal cycling profile.

The TruSeq® DNA PCR-Free Sample Preparation Kit (Illumina, USA) was used to create sequencing libraries in accordance with the manufacturer’s instructions. The quality of the libraries was assessed using the Qubit® 2.0 Fluorometer from Thermo Fisher Scientific and the Agilent Bioanalyzer 2,100 system. Sequencing was conducted on an Illumina NovaSeq platform, producing 250 base pair paired-end reads. The FLASH algorithm was used to merge the paired-end reads.

Quality filtering of the raw sequences was conducted using QIIME (Version 1.9.1) to generate high-quality clean tags. The UCHIME algorithm was then applied to remove chimeric sequences, resulting in the acquisition of effective tags. The operational taxonomic units (OTUs) were created by grouping sequences with ≥97% similarity using the UPARSE algorithm (Version 7.0.1001). The Silva 16S rRNA Database was used to annotate the OTUs taxonomically. To guarantee sample comparability, the OTU abundance was normalized using the sample with the lowest sequence count.

Alpha diversity analysis was undertaken by Mothur. Beta diversity was assessed using partial least squares discriminant analysis (PLS-DA) and principal coordinate analysis (PCoA) of the Jaccard distance. Jaccard-based Adonis test was analysis to calculate statistical differences between groups. The effect size (LEfSe) of linear discriminant analysis (LDA) was employed to distinguish between particular bacterial species in various groups.

### 2.9 Gas chromatography–mass spectrometry (GC–MS)

SCFAs including acetic acid, propionic acid, butyric acid, isobutyric acid, valeric acid, and isovaleric acid were detected by GC–MS. 30 mg of fecal samples were weighed into 2.5 mL centrifuge tubes, and Millipore water was added to reach a total weight of 300 mg. The samples were then homogenized with steel beads (60 Hz, 30 s on, 15 s off, repeated for 30 s). 600 μL of acetone was added, vortexed and then, 30 μL of 50% sulfuric acid, 600 μL of diethyl ether, 90 mg of sodium chloride, and 90 mg of activated charcoal were incorporated and vortexed for an additional 2 min. The mixture was centrifuged at 4 °C for 3 min at 12,000 rpm. After collecting and filtering the upper organic phase through a 0.22 μm membrane, the first two filtrate drops were discarded. The remaining filtrate was collected and analyzed using an Agilent 7,890–5977B GC–MS platform fitted with a DB-FFAP column (30 m × 0.25 mm × 0.25 μm). The actual content of SCFAs in the samples was calculated using Mass Hunter quantification software through calibration with standard curves.

### 2.10 Immunohistochemistry (IHC)

Immunohistochemistry was used to examine the expression of hepatic E-cadherin, N-cadherin, MMP9, and VEGF. After being dewaxed in xylene, paraffin-embedded tissues were rehydrated using an ethanol gradient elution. The slides were repaired in sodium citrate buffer before being blocked with a 3% BSA solution for 30 min. After that, primary antibodies such as E-cadherin (1:400, Boster, China), N-cadherin (1:400, Boster, China), MMP9 (1:400, Boster, China), and VEGF (1:400, Boster, China) were added to the slides and incubated at 4 °C for the entire night. The signals were generated using the MaxVision™ HRP-Polymer anti-Rabbit IHC kit from Maxim, China, along with DAB.

Using a digital tissue section scanner, immunohistochemical slides were scanned, and the Servicebio image analysis system was used to quantify the tissue measurement areas. Based on staining intensity, the marker expression was divided into four grades: 0 being negative (no coloration), 1 being weakly positive (light yellow), 2 being moderately positive (brownish yellow), and 3 being strongly positive (tan). By multiplying the percentage of each intensity level by the corresponding score, the H-score was determined: H-score = (percentage of weak intensity area × 1) + (percentage of moderate-intensity area × 2) + (percentage of strong intensity area × 3).

### 2.11 RNA extraction and quantitative real-time PCR (qPCR)

Total RNA was extracted from frozen liver tissues using TRIzol reagent (Invitrogen, USA) according to the manufacturer’s protocol. The concentration and purity of the RNA were determined using a NanoDrop2000 spectrophotometer (Thermo Fisher Scientific, USA). Subsequently, 1 μg of total RNA was reverse-transcribed into cDNA using the HiScriptR III RT SuperMix for qPCR Kit (Vazyme, China). qPCR was performed on a QuantStudio™ 3 System (Thermo Fisher Scientific, USA) using SYBR Green Master Mix (Vazyme, China). The relative mRNA expression levels of FFAR2 and FFAR3 were calculated using the 2^−ΔΔCt^ method and normalized to the housekeeping gene GAPDH. The primer sequences are provided in Supplementary Table S2.

### 2.12 Western blot analysis

Protein expression of TGF-β and Smad4 was analyzed by Western blot (WB). Liver tissue were homogenized in RIPA buffer supplemented with 1% protease/phosphatase inhibitors, followed by centrifugation (12,000 g, 5 min, 4 °C). Supernatant proteins were resolved on 10% SDS-PAGE gels and electro-transferred to PVDF membranes. After 2 h blocking with 5% BSA, membranes were probed overnight at 4 °C with primary antibodies against TGF-β (1:1,000; Boster, China) and Smad4 (1:1,000; Boster, China). Subsequently, the membrane was incubated with anti-rabbit IgG antibodies (1:100,000, Abcam, UK) for 2 h at room temperature. Chemiluminescent signals were captured using an imaging system, with band intensities quantified via ImageJ software (National Institutes of Health, USA) after normalization to β-actin (1:1,000; Boster, China).

### 2.13 Statistical analysis

For comparisons among multiple groups, one-way ANOVA followed by Tukey’s *post-hoc* test for multiple comparisons was used for normally distributed data. The Kruskal–Wallis or Mann–Whitney U tests followed by Dunn’s *post-hoc* test was used for non-parametric data. The chi-square test or Fisher’s exact test, as applicable, were used to assess categorical data. Spearman’s rank correlation coefficient was used to assess the relationship between the relative abundance of specific bacterial genera and fecal SCFA concentrations. The linear discriminant analysis (LDA) effect size (LEfSe) method used its internal statistical tests with a set threshold of LDA score >3.0. GraphPad Prism 8.0 (GraphPad, San Diego, CA, USA) was used to perform statistical analyses and creating graphs. *p*-values below 0.05 were regarded as statistically significant.

## 3 Results

### 3.1 Clinical characteristics of the enrolled participants

A total of 97 individuals were enrolled in this study, comprising 39 healthy individuals (H group), 21 postoperative metastases patients of CRC (M group), and 37 postoperative non-metastases patients of CRC (C group). The sample size was determined based on the number of qualified patients that could be recruited within a specific period. The characteristics of the three groups are presented in Table 1. There were no significant differences in the age, sex, body mass index (BMI), smoking history, and alcohol use among the three groups (*p* > 0.05), and primary tumor location as well as pathological stage were not significantly different between the M and C groups (*p* > 0.05).

**Table 1:** Clinical characteristics of health people and postoperative metastasis and non-metastasis patients of CRC.

Characteristics		Postoperative metastases patients of CRC (M, *n* = 21)	Postoperative non-metastases patients of CRC (C, *n* = 37)	Health people (H, *n* = 39)	*p*
Demographic characteristics					
Age (years)		63 ± 7	61 ± 9	60 ± 7	0.377
BMI (kg/m^2^)		23.85 ± 2.93	22.76 ± 2.29	23.61 ± 2.70	0.224
Gender	Male	15	22	20	0.317
	Female	6	15	19	
Smoking	Yes	13	19	15	0.201
	No	8	18	24	
Alcohol use	Yes	13	22	23	0.975
	No	8	15	16	
Pathological characteristics					
Pathological stage	II	7	18	–	0.258
	III	14	19	–	
Primary tumor location	Colon	12	21	–	0.977
	Rectum	9	16	–	

Values are mean ± SD. Categorical data are described by numbers. The Chi-square test or Fisher’s exact test were used to assess categorical data. One-way ANOVA followed by Tukey’s *post-hoc* test for multiple comparisons was used for normally distributed data. BMI, body mass index; CRC, colorectal cancer.

Levels of TGF-β, IL-6, IL-10, and IL-17 in serum were assessed to evaluate variations in CRC-related inflammatory and immune cytokines (Table 2). Compared to the H groups, postoperative patients of CRC had higher levels of IL-6 (M vs. H, *p* = 0.017; H vs. C, *p* = 0.038). Conversely, the IL-10 levels in M group was significantly lower than that in C and H groups (M vs. H, *p* < 0.001; M vs. C, *p* = 0.029). TGF-β levels were significantly higher in the M group compared to the C group (*p* = 0.036), while there was no statistically difference when compared to H group (*p* > 0.05). Interestingly, TGF-β levels were significantly lower in the C group compared to the H group (*p* = 0.011), indicating a bidirectional regulation effect on postoperative metastasis. IL-17, an effector cytokine of Th17 cells, exhibited no significant differences across the three groups.

**Table 2:** Cytokines concentration in serum of health people and postoperative metastasis and non-metastasis patients of CRC.

Cytokines	Postoperative metastases patients of CRC (M, *n* = 16)	Postoperative non-metastases patients of CRC (C, *n* = 24)	Health people (H, *n* = 38)	*p*
TGF-β (pg/mL)	16.39 ± 9.16^b^	13.31 ± 5.66^a^	18.54 ± 8.20^b^	0.007
IL-6 (pg/mL)	5.92 ± 1.78^a^	5.39 ± 1.24^a^	4.68 ± 0.88^b^	0.005
IL-10 (pg/mL)	3.31 ± 1.90^a,b^	5.79 ± 2.56^a^	8.34 ± 3.55^b^	<0.001
IL-17 (pg/mL)	13.33 ± 2.50	13.81 ± 2.32	13.41 ± 3.14	0.441

Values are mean ± SD. Statistical significance was determined by one-way ANOVA followed by Tukey’s *post-hoc* test. ^a^Denotes significant differences compared to the H group with *p* < 0.05. ^b^Denotes significant differences compared to the C group with *p* < 0.05.

### 3.2 Distinct fecal microbiota characteristics in postoperative metastasis and non-metastasis patients of CRC

**Figure 2:**
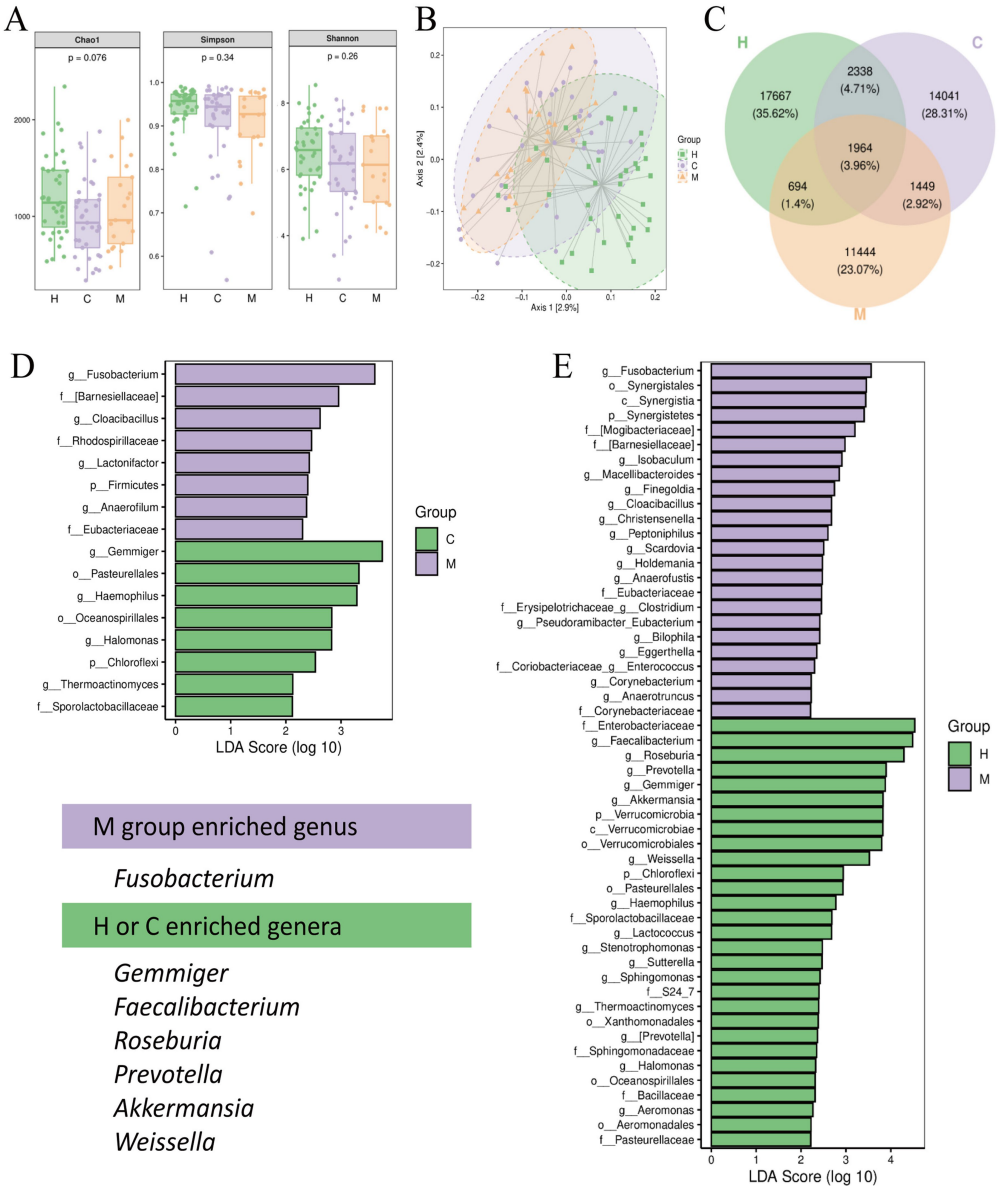
Distinct fecal microbiota characteristics in postoperative metastasis and non-metastasis patients of CRC. **(A)** Alpha-diversity analysis of gut microbiota via Chao1, Simpson, and Shannon indices; **(B)** principal coordinate analysis (PCoA) of β-diversity based on Jaccard distance matrix; **(C)** Venn diagram outlined the number of common and unique overlapping operational taxonomic units (OTUs) of three groups; **(D,E)** LDA scores of differentially abundant taxa between C and M, and H and M using LEfSe (LDA > 3). H: Health people group (*n* = 39); C: postoperative non-metastases patients of CRC (*n* = 37); M: postoperative metastases patients of CRC (*n* = 21). ^*^p < 0.05, ^**^p < 0.01, ^***^p < 0.001.

To investigate whether there were differences in gut microbiota characteristics among the postoperative patients of CRC, fecal samples from all the participants were analyzed for 16 s rRNA sequencing. The Chao1, Shannon, and Simpson indices had no significant differences among the three groups (*p* > 0.05, Figure 2A).

The PCoA based on the Jaccard distance, which measures the dissimilarity between samples, was performed to evaluate β-diversity. There were distinct separations between the M and H groups, indicating significant differences in their microbial community compositions (Figure 2B). The C group was also distinguishable from the H group but is relatively closer, suggesting a more similar microbial profile to the H group when compared to the M group (Figure 2B). The Jaccard-based Adonis test results verified the significant differences in microbial community composition among the three groups (*F* = 1.498, *p* < 0.01). The Venn diagram illustrated both the common and distinct overlapping OTUs among three groups; among them, the H group shared 4,302 OTUs with the C group and 2,640 with the M group, and the C and M groups shared 3,412 OTUs (Figure 2C), indicating that there was a greater overlap and similarity in OTU composition between the C and H groups than between the M and H groups.

**Figure 3:**
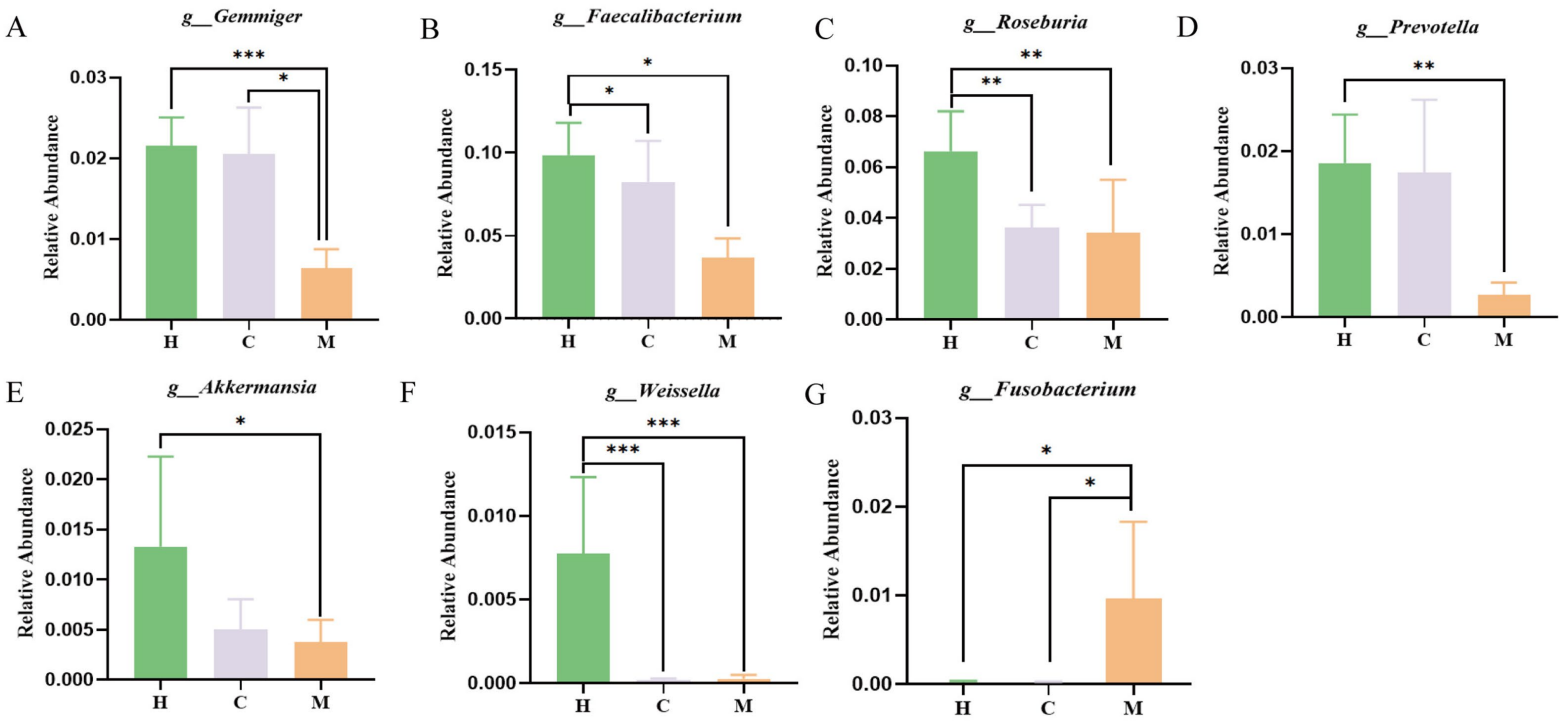
The relative abundance of seven differential bacterial genera among the three groups. **(A–G)** The relative abundance of *Gemmiger*, *Faecalibacterium*, *Roseburia*, *Prevotella*, *Akkermansia*, *Weissella,* and *Fusobacterium*, respectively. Values are expressed as mean ± SEM; the Kruskal–Wallis test followed by Dunn’s *post-hoc* test was used for non-parametric data. H: Health people group (*n* = 39); C: postoperative non-metastases patients of CRC (*n* = 37); M: postoperative metastases patients of CRC (*n* = 21). ^*^p < 0.05, ^**^ *p* < 0.01, ^***^p < 0.001.

Moreover, LEfSe was employed to further distinguish between particular taxa of bacteria in various groups (LDA > 3). In the LefSe analysis between the M group and the H group, the *Verrucomicrobia*, *Verrucomicrobiae*, *Verrucomicrobiales*, *Enterobacteriaceae*, and *Akkermansia*, *Weissella*, *Gemmiger*, *Prevotella*, *Roseburia*, and *Faecalibacterium* were significantly enriched in the H group; the M group exhibited a significant enrichment of *Synergistetes*, *Synergistia*, *Synergistales*, *Mogibacteriaceae*, and *Fusobacterium*. In the LefSe analysis between the M and C groups, the *Pasteurellales*, *Haemophilus* and *Gemmiger* were significantly enriched in the C group, while the *Fusobacterium* was notably enriched in the M group. A further focus at the genus level revealed that there were six differentially abundant genera enriched in the H or C groups, including *Gemmiger*, *Faecalibacterium*, *Roseburia*, *Prevotella*, *Akkermansia*, and *Weissella.* Among them, the genus *Gemmiger* was significantly enriched in both H and C groups. LEfSe analysis identified one genus, *Fusobacterium*, that was significantly enriched in the M group compared to the C group (Figures 2D,E). The relative abundance of seven differential bacterial genera among the three groups was presented in Figure 3.

### 3.3 Depletion of fecal SCFAs in postoperative metastasis patients of CRC

**Figure 4:**
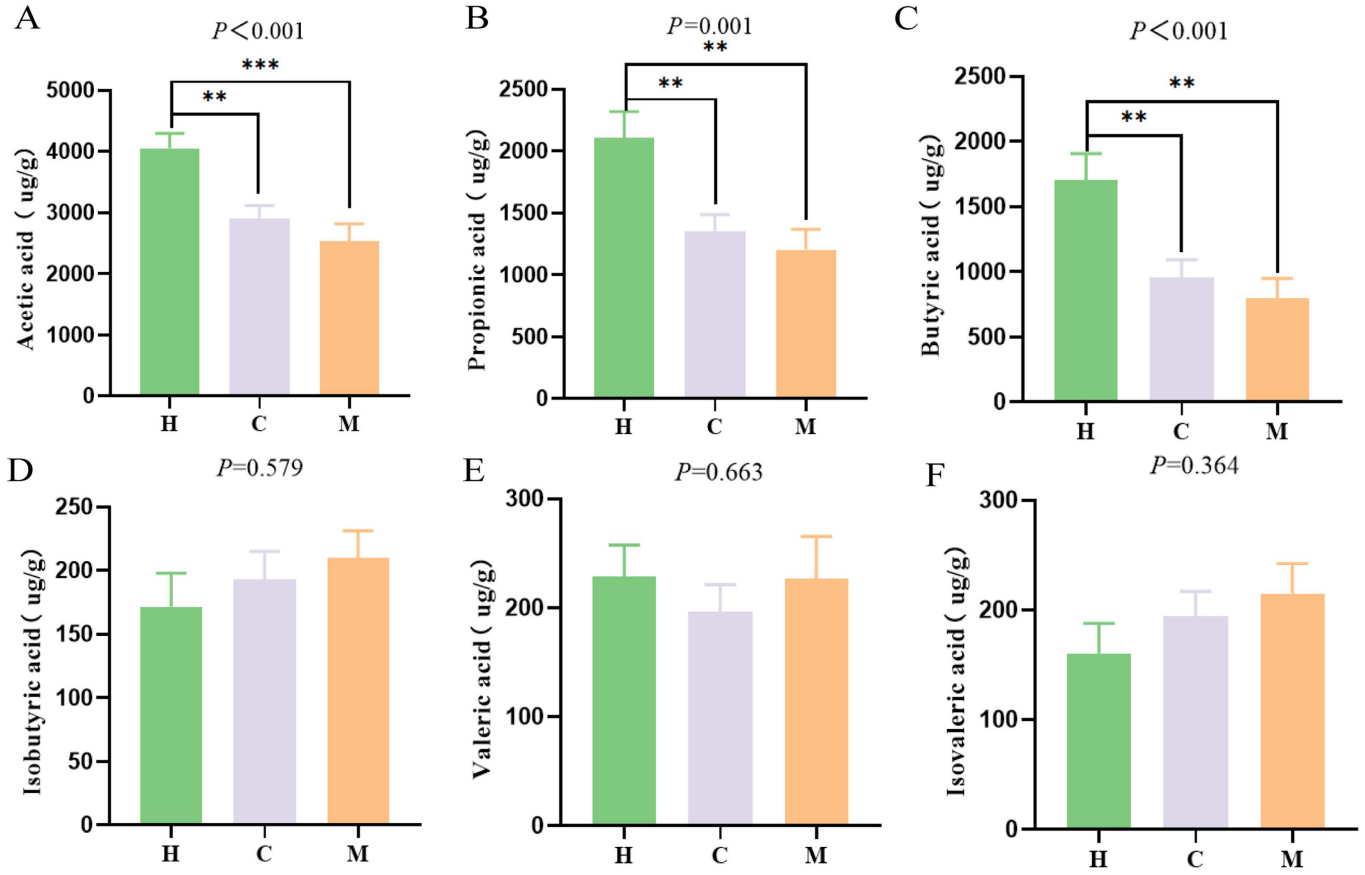
Difference of short-chain fatty acids (SCFAs) in feces among three groups. **(A–F)** The content of acetic, propionic, butyric, isobutyric, valeric, and isovaleric acid, respectively. Values are expressed as mean ± SEM. Statistical significance was determined by one-way ANOVA followed by Tukey’s *post-hoc* test. H: Health group (*n* = 39); C: postoperative non-metastases patients of CRC (*n* = 37); M: postoperative metastases patients of CRC (*n* = 21). *p < 0.05, **p < 0.01, ***p < 0.001.

SCFAs are recognized as health-promoting metabolites that are known to be produced by the beneficial bacteria in the gut. The genera that were significantly enriched in the healthy participants (H group) or the postoperative non-metastatic group of CRC (C group), such as *Gemmiger*, *Faecalibacterium*, and *Akkermansia*, have been reported to be associated with the production of SCFAs. Therefore, we employed GC–MS to further examine the concentrations of the six most common SCFAs in feces (Figure 4). According to the results, the levels of acetic, propionic, and butyric acid in the three groups varied significantly (*p* < 0.001, Figures 4A–C). Specifically, the H group had significantly higher levels of acetic, propionic, and butyric acids compared to both the M and C groups (*p* < 0.05). Notably, although the C group exhibited higher concentrations of butyric, propionic, and acetic acids compared to the M group, the differences were not statistically significant (*p* > 0.05), which may be due to the complexity of metabolite profiles in human samples, so more sample sizes are needed for further verification. To directly link the observed microbial shifts to metabolic changes, we performed a correlation analysis between the key differential bacterial genera and SCFA concentrations (Supplementary Figure S4). The analysis revealed that the relative abundance of *Fusobacterium* was significantly and negatively correlated with fecal butyrate concentration (*r* = −0.247, *P_adj_* < 0.05). In contrast, the abundance of *Gemmiger s*howed a significant positive correlation with butyrate levels (*r* = 0.253, *P_adj_* < 0.05). These findings suggest that the dysbiosis in metastatic patients directly contributes to the depletion of protective SCFAs. Therefore, we next verified this hypothesis in animal models. Therefore, we next verified this hypothesis in animal models.

### 3.4 Effects of FMT on tumor progression in an orthotopic CRC model mice

To verify the biological effects of feces microbiota from postoperative patients of CRC, FMT was conducted in antibiotic-treated orthotopic transplantation model mice of CRC. Figure 1A illustrates the experimental process. A total of 60 mice underwent surgery, and 48 mice successfully detected bioluminescence using the PerkinElmer IVIS Spectrum Imaging System 1 week after surgery. Twelve mice were excluded from the study due to failure to recover from surgery (4 mice), absence of abdominal bioluminescence (5 mice), or low body weight (below 18 g; 3 mice). The remaining 48 mice were divided into 4 groups (12 mice per group) based on body weight. The groups received FMT from the metastasis (FMT-M), non-metastasis (FMT-C), or healthy (FMT-H) human donors, or normal saline as a control (FMT-NS), respectively, and were subjected to corresponding interventions.

After 5 weeks of intervention, the FMT-NS (8/12, 66.7%) and FMT-M groups (7/12, 58.3%) had a noticeably greater number of deceased mice when compared to the other two groups (Table 3). According to imaging *in vivo* with the IVIS Spectrum system, the fluorescence areas in the FMT-C and FMT-H groups were visually smaller than those of the FMT-NS and FMT-M groups (Figure 1B). Histological examination of liver tumors confirmed that the FMT-H and FMT-C groups exhibited significantly reduced tumor metastasis compared to the FMT-M and FMT-NS groups (Figure 1B). However, the proportion of mice with metastases had no significant difference among the four groups by combining in vivo IVIS imaging and liver tumor histology to assess metastasis in mice (*p* > 0.05, Table 3). After intervention, FMT-C group exhibited a significant decrease in cecal tumor weights compared to FMT-M group (*p* < 0.05, Figure 1C). The FMT-H group demonstrated significantly reduced in liver weights when compared to the FMT-M and FMT-NS groups (*p* < 0.05; Figure 1D). Overall, the FMT-M group exhibited varied degrees of promoting effect in mortality, cecal tumor size, and liver metastasis compared to the FMT-H group, suggesting that fecal microbiota from postoperative metastatic CRC patients may exert a certain promoting effect on tumor progression in orthotopic CRC mouse models. Conversely, the fecal microbiota from postoperative non-metastatic CRC patients reversed these changes in the model mice.

**Table 3:** Number (proportion) of death and metastasis in the CRC orthotopic transplantation model mice after FMT.

Groups	Total number	Number of deaths (proportion, %)	Number of metastasis (proportion, %)
FMT-NS	12	8(66.67)	5 (41.67)
FMT-M	12	7 (58.33)	5 (41.67)
FMT-H	12	3 (25.00)	3 (25.00)
FMT-C	12	2 (16.67)	4 (33.33)
*p* value	–	0.041*	0.909

Categorical data are described by numbers. The Chi-square test was used to assess categorical data. *p < 0.05 was considered statistically significant.

### 3.5 Gut microbiota and SCFAs profile in model mice after FMT

**Figure 5:**
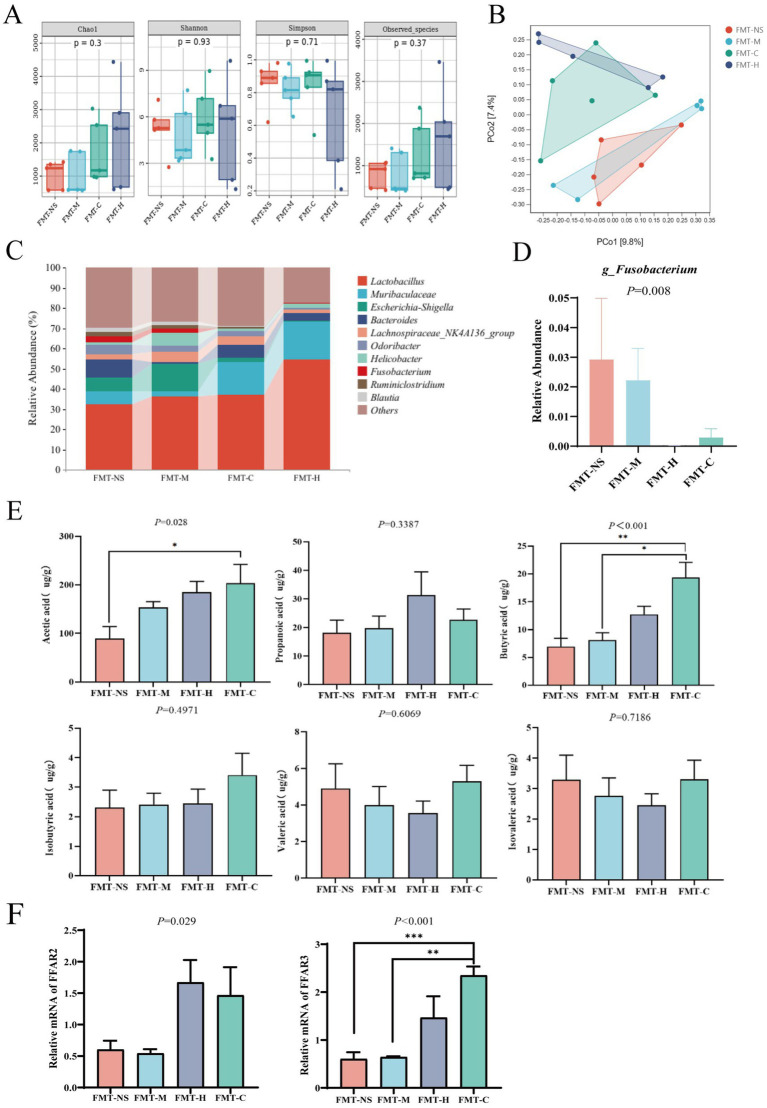
The difference of gut microbiota in model mice receiving FMT from different groups. **(A)** Chao1, Simpson, and Shannon indexes, and observed-species for alpha-diversity analysis of gut microbiota; **(B)** principal coordinate analysis (PCoA) of β-diversity based on Jaccard distance matrix; **(C)** the histogram of relative abundance of the top 10 bacterial genera; **(D)** difference of the relative abundance of *g_Fusobacterium* among four groups; **(E)** difference of six most prevalent short-chain fatty acids (SCFAs) in feces among four groups, involving acetic, propionic, butyric, isobutyric, valeric, and isovaleric, respectively; **(F)** relative mRNA expression of FFAR2 and FFAR3 in liver tissue by qPCR. Values are expressed as mean ± SEM (*n* = 6 per group); the Kruskal–Wallis test followed by Dunn’s *post-hoc* test was used for non-parametric data. *p < 0.05, **p < 0.01.

We collected stool samples from model mice after FMT and performed 16S rRNA sequencing analysis to evaluate the effect of FMT on gut microbiota in model mice. The Chao1, Shannon and Simpson indices as well as observed-species, did show any significant differences across the four groups, indicating no substantial variation in bacterial richness and evenness (Figure 5A). Furthermore, the PCoA anlaysis and Jaccard-based ANOSIM test revealed pronounced distinctions in microbial composition between the FMT-C and FMT-M groups (Figure 5B; Supplementary Table S1). In contrast to the FMT-M group, the FMT-C group was notably closer to the FMT-H group. A significant difference in *Fusobacterium* was observed when the relative abundance of the top 10 bacterial genera was compared across the four groups (Figure 5C; *p* = 0.008). *Fusobacterium* abundance was significantly higher in the FMT-NS and FMT-M groups, but no significant differences were found when compared to the FMT-H and FMT-C groups.

In addition to fecal microbiota analysis, we also assessed SCFAs in the stool samples of model mice, indicating that the level of butyric acid was markedly elevated in the FMT-C group in comparison to the FMT-M group (Figure 5D; *p* < 0.01). An apparent elevation in acetic acid levels was detected in both the FMT-C and FMT-H groups, with the FMT-C group showing the highest levels. However, there was no statistically significant difference in acetic acid between the FMT-M and FMT-C groups (Figure 5E).

To explore how SCFAs might exert their effects within the liver, we measured the mRNA expression of their key receptors, FFAR2 and FFAR3, in liver tissues. qPCR analysis revealed that the mRNA expression of both FFAR2 and FFAR3 was significantly downregulated in the FMT-M group compared to the FMT-H and FMT-C groups (Figure 5F; *p* < 0.05). This suggests that the systemic SCFAs impair may be mediated through both key SCFA receptor in the liver, thereby affecting the progression of metastasis.

### 3.6 Impact of FMT on EMT progression of model mice

**Figure 6:**
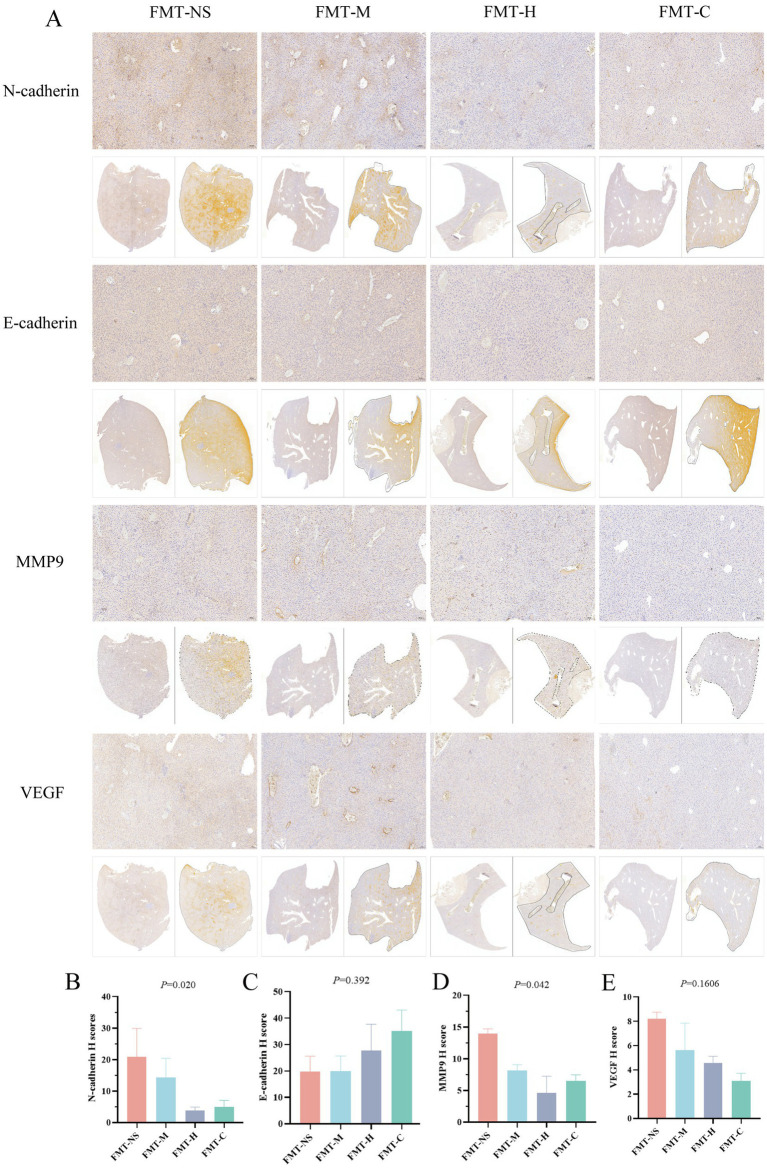
Comparison of liver EMT-related protein expression via immunohistochemistry in four groups. **(A)** Representative immunohistochemical staining of N-cadherin, E-cadherin, MMP-9, and VEGF in livers (Scale bar = 100 μm); **(B)** the histochemistry score (H-score) of N-cadherin; **(C)** the H-score of E-cadherin; **(D)** the H-score of MMP-9; **(E)** the H-score of VEGF. Values are expressed as mean ± SEM (*n* = 3 per group); The Kruskal–Wallis test followed by Dunn’s *post-hoc* test was used for non-parametric data.

The epithelial-mesenchymal transition (EMT) cascade constitutes a pivotal mechanism in tumor metastasis, with E-cadherin, N-cadherin, VEGF, and MMP-9 representing essential molecular markers involved in this biological process. In this study, we employed immunohistochemistry to assess the EMT progression in model mice among the three groups. FMT-NS and FMT-M groups demonstrated significantly higher numbers of brown-stained hepatic N-cadherin-positive cells compared to FMT-H and FMT-C groups (Figure 6A), with quantitative H-scores showing the lowest N-cadherin expression in FMT-H followed by FMT-C (Figure 6B). Conversely, E-cadherin exhibited reduced expression in the FMT-NS and FMT-M groups when compared to the FMT-H and FMT-C groups (Figure 6C), while MMP-9 showed elevated levels in the FMT-NS and FMT-M groups (Figure 6D). VEGF expression exhibited highest levels in the FMT-NS group followed by the FMT-M group, although there were no significant differences among the four groups (Figure 6E). This results indicated that fecal microbiota from postoperative metastasis patients of CRC may regulate EMT progress by enhancing N-cadherin and MMP9 levels, thereby promoting the process of metastasis.

### 3.7 Impact of FMT on TGF-β/Smad4 signaling of model mice

**Figure 7:**
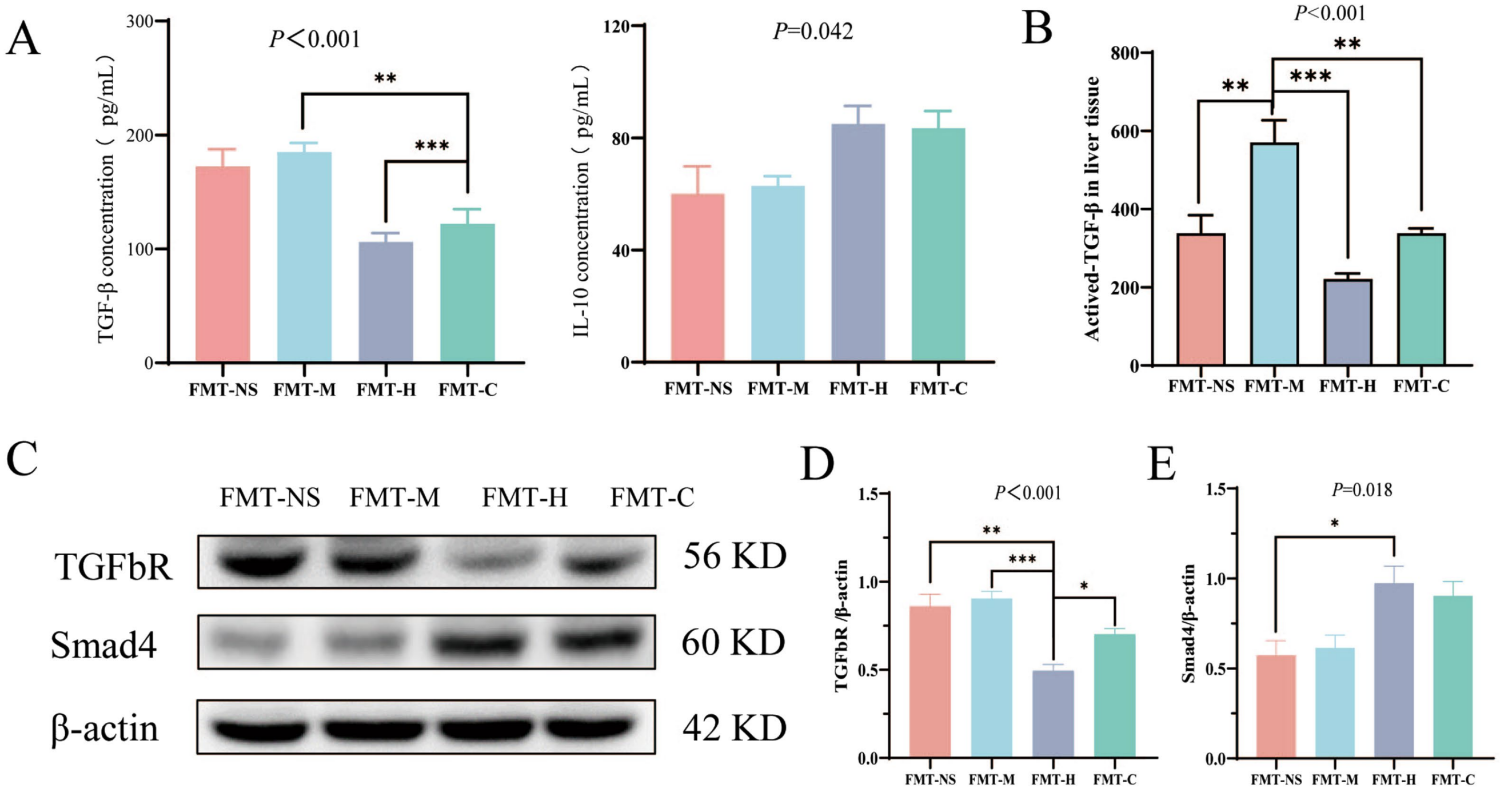
Effects of FMT on TGF-β/Smad4 signaling in an orthotopic CRC mouse model. **(A)** TGF-β and IL-10 concentration of four groups in serum (*n* = 4 per group); **(B)** TGF-β concentration in liver tissue homogenates; **(C–E)** relative protein levels of TGF-β and Smad4 in liver tissue of four groups by western blot (*n* = 3 per group); values are expressed as mean ± SEM. The Kruskal–Wallis test followed by Dunn’s *post-hoc* test was used for non-parametric data. *p < 0.05, **p < 0.01, ***p < 0.001.

As mentioned in this study before, the IL-10 and TGF-β levels in serum were found significantly different between postoperative metastasis and non-metastasis patients of CRC. To further validate the impact of gut microbiota on IL-10 and TGF-β cytokines, serum levels of IL-10 and TGF-β were measured in model mice after FMT, revealing that TGF-β concentrations in the FMT-M group were significantly higher than those in both FMT-H and FMT-C groups (Figure 7A, FMT-M vs. FMT-H, *p* < 0.001; FMT-M vs. FMT-C, *p* = 0.005). To further validate this finding at the tissue level, we measured TGF-β concentrations in liver homogenates. The results showed that hepatic TGF-β levels were markedly elevated in the FMT-M group compared with the FMT-H and FMT-C groups (Figure 7B; *p* < 0.001), consistent with the serum data. Furthermore, protein expression of hepatic TGF-β was markedly elevated in the FMT-M group compared with the FMT-H group (Figures 7C,D; *p* < 0.001). A trend toward reduced Smad4 protein expression was observed in the FMT-M group when contrasted with the FMT-H group, although this difference did not reach statistical significance (Figures 7C,E; *p* = 0.056). Intriguingly, mice which receiving FMT from postoperative non-metastasis patients of CRC exhibited increased hepatic TGF-β expression accompanied by partial restoration of Smad4 expression.

## 4 Discussion

This study provides the first comprehensive characterization of the gut microbiota and its metabolic profile in the context of postoperative CRC metastasis. Our 16S rRNA sequencing analysis revealed significant disparities in the gut microbial community structure between postoperative metastatic (M) and non-metastatic (C) CRC patients. Notably, the microbiota of non-metastatic patients exhibited a closer resemblance to that of healthy controls (H) than to metastatic patients, as evidenced by β-diversity assessments and OTU overlap. This suggests that a gut microbial profile closer to a healthy state may be associated with a lower risk of postoperative metastasis.

To investigate the functional consequences of these distinct microbial profiles, we performed FMT into an orthotopic CRC mouse model. The results provided compelling evidence that microbiota from metastatic patients (FMT-M) significantly accelerated tumor progression, increased liver metastasis, and heightened mortality compared to microbiota from non-metastatic (FMT-C) or healthy (FMT-H) donors. Conversely, the microbiota from the C and H groups suppressed primary tumor growth and liver tumor weight. Importantly, the gut microbiota structure of the recipient mice mirrored that of their human donors, confirming successful engraftment and validating the functional impact of these distinct microbial communities. These findings align with previous work showing that different stages of CRC are characterized by specific microbiota profiles (Yachida et al., 2019) and extend this concept to the critical postoperative setting.

A deeper analysis of the microbial signatures revealed that the postoperative metastatic phenotype was associated with an enrichment of the pathogenic genus *Fusobacterium* and a depletion of beneficial, SCFA-producing genera, including *Gemmiger*, *Faecalibacterium*, and *Roseburia*. The enrichment of *Fusobacterium*, particularly *F. nucleatum*, in CRC has been extensively documented. Multi-omics studies demonstrate that *F. nucleatum* is enriched in CRC tumor tissues and associated gut microbiota (Feng et al., 2015; Kostic et al., 2012; Ahn et al., 2013; Castellarin et al., 2012), establishing its role as a pathogenic driver of CRC progression (Gopalakrishnan et al., 2018). Moreover, the abundance of *F. nucleatum* increase with tumor malignancy, contributing to the progression of CRC (Yachida et al., 2019). Our findings implicate *Fusobacterium* as a potential driver in the postoperative metastatic cascade, a role further substantiated by its significant negative correlation with fecal butyrate concentrations observed in our patient cohort. Concurrently, the depletion of beneficial genera is equally critical. *Gemmiger*, which was significantly enriched in both non-metastatic and healthy individuals, is a known producer of SCFAs, particularly butyrate (Sang et al., 2022; Li et al., 2021). Our correlation analysis reinforces this functional link, demonstrating a significant positive association between *Gemmiger* abundance and butyrate levels within our study population. Its depletion has been linked to various pathological conditions, and its restoration is associated with beneficial interventions (Liu et al., 2022; Medawar et al., 2021). Thus, we propose that *Gemmiger* may exert beneficial effects in postoperative CRC patients. Similarly, the reduction in *Faecalibacterium* and *Roseburia*—two of the most important butyrate-producers in the human gut (Louis and Flint, 2009; Alcazar et al., 2022; Qian et al., 2022; Nie et al., 2021) with well-established anti-inflammatory and barrier-protective functions (Martín et al., 2014; Martín et al., 2015; Carlsson et al., 2013; Wrzosek et al., 2013; Song et al., 2022; Shen et al., 2022)—further supports the hypothesis that a loss of beneficial microbial functions contributes to a pro-metastatic environment. It follows that the beneficial genera, such as *Gemmiger*, *Faecalibacterium* and *Roseburia*, consumed in postoperative metastasis patients of CRC uniformly exhibit probiotic attributes characterized by SCFAs production capabilities.

The observed shifts in SCFA-producing bacteria were reflected in the metabolic profiles. Patients in the M group exhibited significantly lower fecal concentrations of acetate, propionate, and butyrate compared to healthy controls. While the difference between the M and C human groups did not reach statistical significance—a finding that may be attributable to the limited sample size or high inter-individual variability—the functional importance of SCFAs was clearly demonstrated in our animal model. Mice receiving FMT from non-metastatic patients showed significantly increased butyrate levels compared to the FMT-M group. This suggests that the microbiota from non-metastatic patients retains a greater capacity for SCFA production, which may exert protective effects. This is consistent with a meta-analysis demonstrating that lower SCFA levels are associated with a higher incidence of CRC (Alvandi et al., 2022). Furthermore, our novel finding that the mRNA expression of both key SCFA receptors, FFAR2 and FFAR3, is downregulated in the livers of FMT-M mice points to a crucial receptor-mediated mechanism. These receptors, activated by different SCFAs (Brown et al., 2003), are known to modulate inflammation, cell proliferation, and metabolism (Parada Venegas et al., 2019). The concurrent transcriptional suppression of both FFAR2 and FFAR3 suggests a broad impairment of SCFA sensing within the liver microenvironment. This loss of signaling could disable multiple anti-tumorigenic pathways, such as the suppression of oncogenic signaling cascades demonstrated in hepatocellular carcinoma models (Song et al., 2023; Lau et al., 2024), thereby contributing to a pro-metastatic state.

Mechanistically, our study connects these microbial and metabolic changes to the epithelial-mesenchymal transition (EMT), a critical process for metastasis. In our model, microbiota from metastatic patients promoted EMT, as evidenced by the significant upregulation of N-cadherin and MMP9 in liver tissues. N-cadherin promotes the transition of cells from an epithelial to a mesenchymal phenotype, enhancing their motility and migratory capacity. Elevated expression of MMP9 is linked to poor prognosis in cancer patients (Yoo et al., 2011; Yang et al., 2014). Increased expression of MMP9 protein degrades the extracellular matrix, affects angiogenesis, and promotes tumor infiltration and metastasis, driving the progression of EMT (Bruno et al., 2018). Elevated N-cadherin enhances cell motility, while MMP9 degrades the extracellular matrix, facilitating tumor invasion.

We further explored the upstream signaling pathways and identified the TGF-β/Smad4 axis as a key mediator. Postoperative metastatic patients and FMT-M mice both exhibited elevated systemic levels of TGF-β. While TGF-β can act as a tumor suppressor in early-stage cancer, it is a well-known driver of EMT in advanced disease (Zhao et al., 2020; Yu et al., 2015). Moreover, TGF-β levels are directly and indirectly modulated by the gut microbiota, including microbiota-induced TGF-β production and enhanced activation of TGF-β (Shouval et al., 2014). The link between the gut microbiota and TGF-β is well-established, but the role of microbial metabolites like SCFAs in this specific context is less clear. Although our current data lacks direct evidence, we propose a plausible hypothesis wherein the depletion of SCFAs, a key consequence of the dysbiosis we observed, contributes to the dysregulation of the TGF-β/Smad4 pathway. This is supported by literature indicating that SCFAs, particularly butyrate, can modulate TGF-β signaling to influence cell function (Hu et al., 2022) and exert protective effects in models stimulated with TGF-β1 (Tian et al., 2023). Critically, this functional switch is often dependent on the status of its downstream mediator, Smad4. Prior studies suggest that TGF-β-mediated EMT induction may depend on Smad4 deficiency, as Smad4 inactivation impairs TGF-β-induced apoptosis and tumor-suppressive effects (Levy and Hill, 2005). Consistent with this, we observed a trend toward reduced Smad4 expression in the FMT-M group (*p* = 0.056). We hypothesize that in the postoperative metastatic setting, gut dysbiosis creates a pro-inflammatory environment characterized by high TGF-β. This, combined with impaired Smad4 function, may hijack the TGF-β pathway, shifting it from a tumor-suppressive to a pro-metastatic signaling cascade that drives EMT.

This study has several limitations that should be acknowledged. First, the data from our human study are correlational, and a direct causal relationship cannot be established from these observations alone. Although the FMT animal model provides potential causal clues, further in-depth mechanism studies are still needed to ultimately confirm it. Second, the sample size was relatively modest, which may limit the statistical power to detect smaller differences, as potentially seen in the SCFA analysis between the C and M groups. Third, while our orthotopic transplantation model standardizes surgical stress across all experimental groups by subjecting them to an identical procedure, this design does not permit the isolation of the independent contribution of the surgical event itself to the metastatic process. It effectively treats surgical stress as a controlled constant, ensuring that observed differences are attributable to the FMT intervention, but the interaction between surgery and microbiota remains an area for future investigation. Finally, our study lacks a “rescue” experiment, such as supplementing butyrate to the FMT-M mice, which would be crucial for definitively establishing the causal role of SCFA depletion in metastasis. Future studies should aim to address these limitations with larger, prospective cohorts and more targeted mechanistic experiments, such as investigating the phosphorylation status of Smad2/3 to confirm pathway activation.

In summary, this study is the first to comprehensively link gut microbiota to postoperative metastasis in CRC patients. Our findings demonstrate that a dysbiotic state, characterized by an enrichment of *Fusobacterium* and a depletion of SCFA-producing bacteria, is associated with and functionally contributes to metastatic progression. This process appears to be mediated, at least in part, by the impairment of the TGF-β/Smad4 signaling axis, leading to enhanced EMT. These results provide a strong rationale for developing microbiota-targeted therapies, such as probiotics, prebiotics, or FMT, as novel strategies to prevent or treat postoperative metastasis in CRC patients.

## 5 Conclusion

In conclusion, our findings suggest that gut microbiota dysbiosis, characterized by an increase in *Fusobacterium* and a decrease in SCFA-producing bacteria like *Gemmiger*, is associated with postoperative CRC metastasis. As summarized in our proposed model (Graphical Abstract), this dysbiosis and the resulting depletion of acetate and butyrate may contribute to a pro-metastatic environment by promoting EMT, a process potentially linked to alterations in the TGF-β/Smad4 signaling axis. These findings highlight the potential of targeting the gut microbiota as therapeutic strategies to prevent metastasis in postoperative CRC patients.

## Data Availability

The datasets presented in this study can be found in the NCBI Sequence Read Archive (SRA): http://www.ncbi.nlm.nih.gov/bioproject/1225466 (BioProject ID: PRJNA1225466) and http://www.ncbi.nlm.nih.gov/bioproject/1225497 (BioProject ID: PRJNA1225497).
