# Supplementary material for: Gut microbiota dysbiosis impairs TGF-β/Smad4 signaling to drive postoperative metastasis in colorectal cancer
Source: Front Microbiol. 2025 Dec 15;16:1654227. doi: 10.3389/fmicb.2025.1654227 (PMC12747669; doi:10.3389/fmicb.2025.1654227)
Supplement: Supplementary file 1 [file Supplementary_file_1.docx]

Supplementary Material

# CT-26-H-LUC cell morphology and fluorescence

The infection efficiency was assessed 24 hours post-incubation using an inverted fluorescence microscope. As indicated by the image, 90% of the cells exhibit green fluorescence (GFP), demonstrating a high lentivirus transfection efficiency suitable for subsequent experiments.


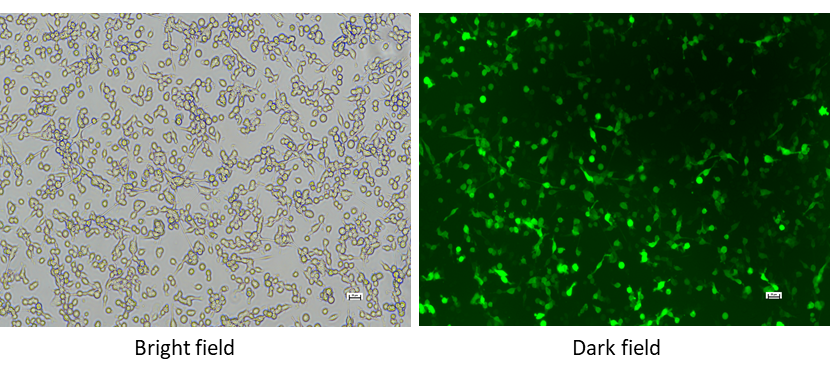


**Supplementary Figure 1.** Observation of CT-26-H-LUC cell morphology and fluorescence under an inverted fluorescence microscope.

# Fecal microbiota smears from model mice before and after antibiotic treatment

The smear (A) before antibiotic intervention shows a variety of Gram-positive and Gram-negative bacteria; in contrast, the smear (B) after intervention reveals almost no bacterial growth. This result suggests that the antibiotic cocktail intervention can eliminate approximately 95% of the bacteria in the gut, successfully creating a relatively sterile intestinal environment.


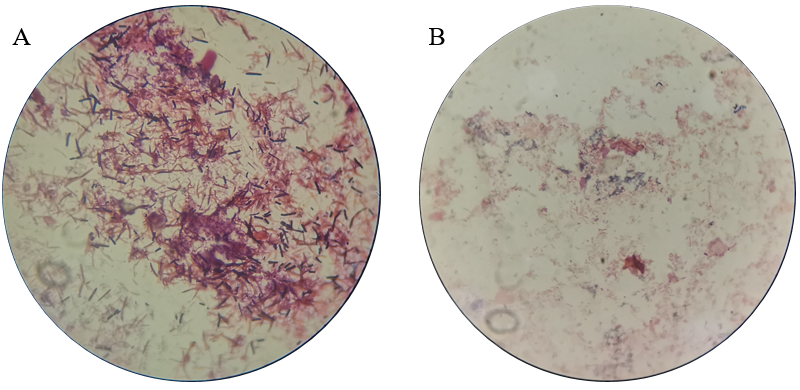


**Supplementary Figure 2.** Fecal microbiota smears from model mice before (A) and after (B) antibiotic treatment. Oil immersion lens: 100X.

# Bioluminescence imaging of CT26-H-LUC cells using the IVIS Spectrum in vivo imaging system

CT26-H-LUC cells were subjected to gradient dilution in a 96-well plate, with 100 μL of cell suspension added to each well at densities of 1×10⁴ cells/mL, 1×10⁵ cells/mL, and 1×10⁶ cells/mL, respectively. Subsequently, 100 μL of D-luciferin potassium salt solution was added to each well, and the luminescence of the cells in the plate was detected using the IVIS Spectrum in vivo optical imaging system. As this figure shown that Luminescence was observed in wells with cell densities of 1×10⁵ cells/mL and 1×10⁶ cells/mL, with more pronounced luminescence in the wells containing 1×10⁶ cells/mL. Therefore, we selected a cell density of 1×10⁶ cells/mL for subsequent cecal cell injections.


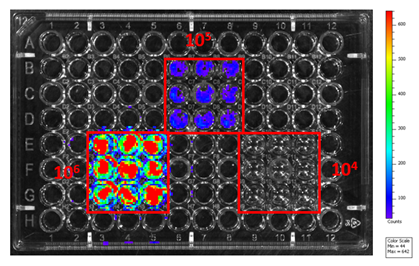


**Supplementary Figure 3.** Bioluminescence imaging of CT26-H-LUC cells using the IVIS Spectrum in vivo imaging system.

# Jaccard-based Anosim test of gut microbiota differences analysis among the four groups in model mice

**Table S1.** Jaccard-based Anosim test of differences analysis among the four groups in model mice.

| Group1 | Group2 | Sample size | Permutations | R | *p*-value | *q*-value | R | *P* |
| --- | --- | --- | --- | --- | --- | --- | --- | --- |
| FMT-NS | FMT-M | 10 | 999 | 0.108 | 0.165 | 0.198 | 0.227 | 0.023* |
| FMT-NS | FMT-C | 10 | 999 | 0.46 | 0.01 | 0.030* |  |  |
| FMT-NS | FMT-H | 10 | 999 | 0.472 | 0.007 | 0.030* |  |  |
| FMT-M | FMT-C | 10 | 999 | 0.18 | 0.136 | 0.198 |  |  |
| FMT-M | FMT-H | 10 | 999 | 0.26 | 0.106 | 0.198 |  |  |
| FMT-C | FMT-H | 10 | 999 | -0.04 | 0.53 | 0.530 |  |  |

^*^*P* < 0.05 means statistically significance.

# Heatmaps of the Spearman’s correlations between the key differentially regulated bacterial species and the SCFAs in clinical study


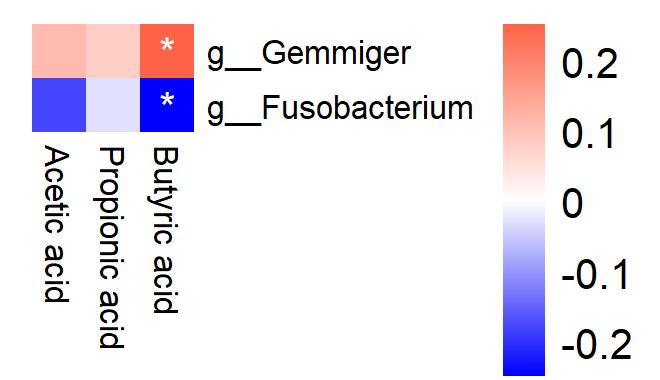


**Supplementary Figure 4.** Heatmaps of the Spearman correlations between the relative abundance of *Fusobacterium* and *Gemmiger* and the quantified levels of butyrate, propionate, and acetate in clinical study (n=97),*Padj < 0.05.
